# DXSP: gesture-only temporal localization of decisive moments in dyadic persuasion

**DOI:** 10.3389/frai.2026.1928901

**Published:** 2026-09-16

**Authors:** Pakpoom Chaimook, Kaoru Sumi

**Affiliations:** Graduate School of Systems Information Science, Future University Hakodate, Hakodate, Japan

**Keywords:** dyadic interaction, gesture recognition, human-AI interaction, persuasive technology, privacy-preserving sensing, skeleton-based analysis, sparse attention, temporal localization

## Abstract

Persuasive systems must detect user engagement in real time in order to adapt their strategy. Existing methods rely primarily on facial expressions or voice signals. Both raise privacy concerns and degrade under low video quality, motion blur, and ambient noise. Body gestures offer a privacy-preserving alternative, since skeleton representations do not encode identifiable facial features. What is missing is a method that localizes the specific moments at which gesture matters, rather than averaging behavior across the whole interaction. We introduce Dyadic Cross-Sparse Pooling (DXSP), a gesture-only neural architecture that localizes sparse decisive moments in dyadic persuasion without using face, voice, or verbal content. DXSP models both participants through cross-attention, weights candidate moments by interpersonal synchrony through a concordance gate, and selects exactly *k* = 3 windows per dyad through a hard top-*k* mechanism. We evaluate DXSP on 46 Japanese dyadic persuasion dialogues using upper-body pose data from the Sapiens Goliath foundation model. First, decisive moments are distributed across the full interaction rather than concentrated in any single phase. A three-component mixture fits the selected positions, but random selection of the same number of windows reproduces this evidence at every value of *k* from 1 to 10. Second, the relative concentration of decisive moments across phases differs between successful and failed outcomes. Successful dyads place 49.1% of their peaks in the opening phase. Failed dyads place 42.0% in the middle phase. Three tests converge on this contrast, including a permutation test that accounts for within-dyad clustering. Only the opening-phase comparison survives correction, and the association is not stable across values of *k*. Third, joint-level differences did not reach significance after correction across the seven joints, and the joint ranking is exploratory. The study contributes a mechanism that returns explicit decisive positions, evidence that mixture-based clustering claims require calibration against a matched null, and a preliminary phase-level link between decisive-moment placement and outcome. Gesture-only sensing therefore relates to persuasion at the level of phase distribution rather than detecting three-phase structure. This offers preliminary guidance for phase-aware intervention timing.

## 1 Introduction

Persuasive technology aims to influence user attitudes or behaviors through computational systems. The effectiveness of such systems depends on detecting user engagement in real time. Without engagement detection, the system cannot adapt its strategy to changing user states. This adaptation requirement creates a need for robust non-verbal sensing in human-AI interaction (Fogg, 2002; Picard, 1997). Recent reviews indicate growing interest in persuasive systems that integrate adaptive behavioral sensing with intelligent agent design (Rogiers et al., 2024).

Existing methods for non-verbal engagement detection rely primarily on facial expressions or voice signals. Facial expression methods analyze action units or learned representations from video frames to infer affective states (Ekman, 1992; Krumhuber et al., 2023). Voice-based methods extract prosodic features such as pitch contour and energy envelope to detect arousal or stance shifts (Schuller et al., 2013). Recent multimodal surveys highlight the rapid expansion of affective computing approaches that combine facial, vocal, and text modalities (Ahmed et al., 2023; Hu et al., 2024; Ramaswamy and Palaniswamy, 2024).

Both facial and vocal modalities face deployment constraints in real-world persuasive systems. Facial expressions become unreliable under low video quality, motion blur, occlusion, or oblique viewing angles. Voice signals degrade under ambient noise, microphone distance variation, or speaker overlap. In addition, both modalities raise privacy concerns. The face uniquely identifies individuals (Krumhuber et al., 2023). The voice carries identity, emotional state, and biometric markers that users may not wish to share with persuasive systems (Schuller, 2018). Recent work in privacy-preserving action recognition explores ways to balance recognition accuracy with identity protection (Kumawat and Nagahara, 2022). These constraints motivate the search for alternative non-verbal signals that are robust to sensing noise and free from identity disclosure.

Body gestures offer such an alternative. Modern pose estimation systems extract skeletal joint positions accurately even under degraded video conditions (Khirodkar et al., 2024). Skeleton representations do not encode identifiable facial features. They preserve interactional dynamics while reducing privacy risk (Gu et al., 2023). Gesture analysis therefore represents a promising direction for persuasive technology.

The most relevant prior work on gesture-based behavioral analysis falls into three categories. The first category analyzes individual gesture trajectories using aggregate statistics across an entire interaction (Burgoon et al., 2016). These methods compute summary features such as mean velocity, total motion energy, or gesture frequency. The second category examines interpersonal synchrony between two individuals using cross-correlation, dynamic time warping, or phase-locking metrics (Tschacher et al., 2014; Wallot et al., 2017). Recent work extends this line by examining multimodal synchronization between voice and gesture (Fauviaux et al., 2024). The third category applies deep learning architectures such as graph convolutional networks or transformers to skeleton sequences for action recognition (Yan et al., 2018; Plizzari et al., 2021; Song et al., 2023; Lee et al., 2023; Shaikh et al., 2024). These methods predict labels from learned spatiotemporal features.

Three limitations emerge from this prior work. First, aggregate statistics dilute the contribution of brief but informative episodes. The temporal location of decisive moments is lost when behavior is averaged across the entire interaction. Second, synchrony metrics measure global coordination but do not identify the specific moments at which coordination matters most for outcome. Third, action recognition architectures are typically designed for short, well-segmented clips rather than for the long, conversational structure of persuasive dyadic interaction. Recent work on dyadic engagement prediction begins to address these gaps using attention-based architectures, but does not explicitly localize decisive moments (Li et al., 2023; Funk et al., 2024).

A complementary theoretical perspective on persuasive interaction comes from Rhetorical Structure Theory (Mann and Thompson, 1988). This framework characterizes persuasive discourse as a hierarchical organization of nucleus and satellite units. Persuasive arguments typically unfold in three positional phases. The opening phase establishes rapport and frames the topic. The middle phase contains the core argumentation. The closing phase consolidates agreement. If gestures track this rhetorical structure, then decisive gesture episodes should cluster at these three positions rather than distribute uniformly across the interaction.

The intersection of these observations suggests a specific computational hypothesis. A model that explicitly localizes a small number of decisive gesture moments may capture persuasion-relevant structure that aggregate behavioral baselines miss. The problem addressed in this study is therefore the following. Given only upper-body skeleton sequences from both members of a dyad, can a model localize the moments that matter for persuasion, and does the temporal placement of those moments relate to the outcome of the interaction? The scope is deliberately narrow in three respects. We use gesture alone and exclude face, voice, and verbal content. We work with low-stakes dyadic dialogues of approximately 2 min recorded under controlled conditions. We treat persuasion outcome as a dyad-level self-report rather than as a behavioral measure. The aim is to characterize the temporal placement of decisive moments rather than to build a classifier of persuasion success.

This study introduces Dyadic Cross-Sparse Pooling (DXSP). DXSP is a gesture-only neural architecture that identifies sparse decisive moments in dyadic persuasive interaction by jointly modeling both participants. The architecture combines three components. Cross-attention captures the dependency of one participant gesture context on the other. A concordance gate weights candidate moments by the degree of interpersonal synchrony in latent space. A hard top-*k* attention mechanism enforces structural sparsity by selecting exactly *k* = 3 windows per dyad. This design reflects the hypothesis that persuasion may depend on a limited set of decisive episodes rather than on cumulative motion. DXSP differs from skeleton-based action recognition architectures in what it optimizes. ST-GCN (Yan et al., 2018), CTR-GCN (Chen et al., 2021), BlockGCN (Zhou et al., 2024), SkateFormer (Do and Kim, 2025), and ProtoGCN (Liu et al., 2025) refine the spatial graph over joints and pool over time to classify a trimmed clip. DXSP treats the dyad as a variable-length window sequence and imposes sparsity along the temporal axis instead, selecting a fixed number of windows rather than weighting all of them.

This study makes three contributions. First, we introduce a hard top-*k* attention mechanism that enforces structural sparsity by construction. Prior soft-attention models encourage sparsity through entropy or L1 penalties without guaranteeing it, and skeleton-based action recognition architectures pool over the whole clip. Neither returns an explicit set of decisive positions. DXSP returns exactly *k* window positions per dyad, which is what makes the phase-level analysis in this paper possible. Second, we show that mixture-based evidence for temporal clustering in sparse-attention models requires calibration against a matched random-selection null. Without this calibration, a Bayesian Information Criterion computed on model-selected positions indicates multi-modal structure that random selection under the same sparsity constraint reproduces. We report this as a negative methodological result that applies to any model of this family. Third, we report preliminary evidence that the relative concentration of decisive moments across phases differs between successful and failed persuasion outcomes, even though no architecture we tested predicts outcome above chance. We interpret this contrast as a positional content distinction. DXSP relates to when persuasion-relevant moments occur rather than to the final outcome.

## 2 Materials and methods

Figure 1 presents an overview of the experimental setup and data collection pipeline used in this study.

**Figure 1:**
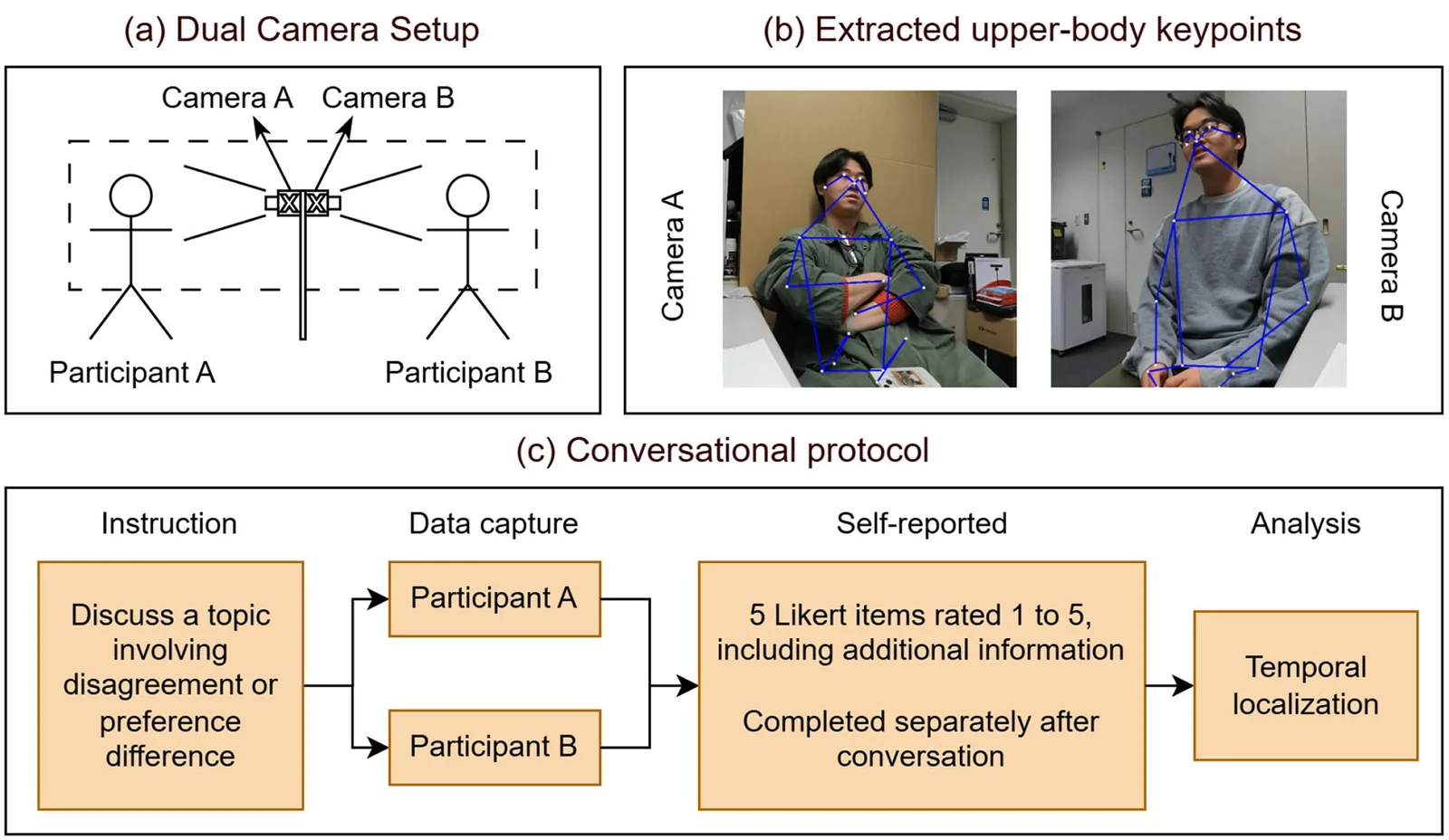
Experimental setup and data collection pipeline. **(a)** Dual camera setup with two participants and two GoPro Hero 10 cameras. **(b)** Seven upper-body keypoints extracted from each camera view. **(c)** Conversational protocol: participants were asked to discuss a topic involving disagreement or preference difference, without assigning explicit persuader or persuadee roles.

### 2.1 Participants and recruitment

We collected a corpus of dyadic persuasion dialogues at Future University Hakodate during December 2025 and January 2026. Participants were recruited through campus announcements and word-of-mouth referral. All participants were university students enrolled at Future University Hakodate. All participants were native Japanese speakers. Participants ranged in age from 18 to 25 years. Both male and female participants were included. Participants were assigned to dyads through scheduling availability rather than by pairing on demographic or personality variables. This naturalistic pairing approach reflects realistic deployment conditions for persuasive AI systems.

According to the ethical review flowchart of Future University Hakodate, this study was determined not to require formal review by the Research Ethics Committee. All participants provided written informed consent before recording. The consent form described the experimental procedure, the use of video and pose data for research purposes, and the right to withdraw without penalty. Participants received a small honorarium for their time. Two forms of privacy should be distinguished. Data collection required video recordings of identifiable participants, which were retained on a secure research server under the terms of the informed consent. Inference operates on a different representation. The analyzed dataset contains only the seven upper-body joint coordinates with face keypoints excluded, and no identifying information beyond participant ID. The privacy-preserving framing of this study therefore refers to what the model receives at inference time, not to the collection stage.

A total of 48 dyads were recorded during the data collection period. Two dyads were excluded from analysis due to incomplete pose extraction caused by camera occlusion during the recording. The final sample for all analyses reported below comprised *n* = 46 valid dyads, corresponding to 92 individual participants.

### 2.2 Experiment protocol

Each recording session consisted of three phases. The first phase was a pre-recording briefing of approximately 5 min. During this phase, an experimenter explained the procedure to both participants. The experimenter described the topic of the upcoming dialogue. The experimenter clarified that the goal was natural conversation rather than persuasive performance. The experimenter did not designate a persuader or persuadee role explicitly. This non-directive design allowed natural role emergence during the interaction.

The second phase was the recorded dialogue itself. Each session was recorded for longer than the analyzed segment. The opening and closing portions of each recording were removed in post-processing so that only the conversation itself was retained. Topics were drawn from a curated set of everyday scenarios. Example topics included restaurant choice for a hypothetical dinner, weekend activity selection for a hypothetical free day, and travel destination preference for a hypothetical vacation. These topics were chosen because they involve genuine personal preference without high real-world stakes. The low-stakes nature reduces social desirability bias while preserving authentic preference expression.

The third phase was a post-recording survey of approximately 3 min. Each participant independently completed a written questionnaire about their subjective experience during the dialogue. The questionnaire included Likert-scale items covering perceived persuasion strength, emotional response, and engagement quality. The questionnaire was administered separately to each participant to prevent influence between partners.

### 2.3 Recording equipment and setup

All dialogues were recorded in a dedicated laboratory room with controlled lighting and minimal background noise. The room measured approximately 4 by 5 meters. Lighting was provided by ceiling-mounted fluorescent fixtures to minimize shadows on participant faces and torsos. Background was a neutral colored wall to minimize visual distractors. Participants were seated facing each other at a separation of approximately 1.5 meters.

Two GoPro Hero 10 cameras were used for video recording. Camera A was positioned to record one participant in upper-body framing from approximately 1.5 meters distance. Camera B was positioned symmetrically to record the other participant. Both cameras recorded at 1080p resolution and 30 frames per second. The framing captured the participant from approximately the waist to slightly above the head, ensuring full visibility of the seven upper-body joints used in subsequent analysis.

Camera synchronization was achieved through a visual cue at the start of each recording. The experimenter clapped hands once visibly in front of both cameras before stepping out of frame to begin the dialogue. The clap was used in post-processing to align the timelines of the two video streams. Audio was not analyzed in this study, but was recorded for potential future multimodal analysis. All video files were transferred to a secure research server immediately after each session.

### 2.4 Self-report measures

The post-session questionnaire included five Likert-scale items measuring distinct affective and motivational responses. Four items measured emotional states corresponding to happiness, sadness, fear, and confusion. The fifth item measured perceived persuasion strength using the question, *How strongly did you feel persuaded during this conversation?* All items were rated on a 1 to 5 scale where 1 indicated minimal experience and 5 indicated very strong experience. The persuasion item served as the primary outcome variable for the present analysis.

Aggregating subjective responses across both participants in a dyad provides a single dyad-level measure of persuasion outcome. We computed the dyad-level outcome score as the arithmetic mean of the persuasion ratings from participant A and participant B. This averaging approach reflects the collaborative nature of the persuasion task. The two participants in a persuasion dialogue mutually shape and respond to each other. A purely individual perspective would obscure this collaborative aspect of the interaction. Two features of the protocol support this choice. First, no persuader or persuadee role was assigned, so influence could flow in either direction or be mutual within a dyad. A single participant rating would capture only one side of an interaction whose direction is not known in advance. Second, the dyad mean matches the unit at which DXSP operates, since the model pools across both participants to produce one prediction per dyad.

We dichotomized the continuous dyad-level outcome score into a binary variable for statistical modeling. Dyads with mean score greater than or equal to 4 were labeled as successful (*n* = 19). Dyads with mean score less than or equal to 3 were labeled as failed (*n* = 27). Dyads with mean score in the open interval (3, 4) would have been excluded as ambiguous, but no dyads fell in this range in our dataset. This dichotomization avoids the difficulties of distributional modeling on a 5-point ordinal scale with small sample size while preserving meaningful outcome variation.

### 2.5 Pose extraction pipeline

Skeletal pose was extracted from each video using the Sapiens Goliath model (Khirodkar et al., 2024). Sapiens Goliath is a recent foundation model for human pose estimation that provides 308 keypoints per frame across the body, face, and hands. The dense representation captures fine detail at the cost of computational expense.

From the full 308-keypoint output, we selected seven upper-body joints for downstream modeling. The selection was guided by three considerations. First, the selected joints support direct comparison with prior work in the same laboratory using the same seven-joint configuration on a parallel Thai corpus. Maintaining the joint configuration isolates the contribution of the model architecture from the contribution of the joint set. Second, the exclusion of face keypoints preserves the privacy-preserving framing of the gesture-only approach. Face keypoints can be used to reconstruct facial geometry sufficient for identification, which would weaken the central design principle of avoiding identity disclosure. Third, the upper-body framing of the recording from approximately the waist to slightly above the head excludes lower-body joints from reliable detection. The seven selected joints are the nose, left shoulder, right shoulder, left elbow, right elbow, left wrist, and right wrist.

The wrist joints require careful attention to the Sapiens Goliath keypoint indexing. The wrist joints are stored inside the hand keypoint blocks of the Sapiens output rather than in the body keypoint block. The left wrist corresponds to index 62 and the right wrist corresponds to index 41 in the Goliath keypoint ordering. The remaining five joints follow standard COCO indexing within the body block. Documenting this indexing supports reproducibility for future work using the same pose model.

Each video frame yielded a (7, 3) array per camera. The three columns represent the x-coordinate, y-coordinate, and detection confidence per joint. The pose extraction was performed offline using a local workstation with an NVIDIA RTX 3070 graphics card. Processing time was approximately 3–5 min per 2-min video at the chosen resolution. This time covers trimming the session to the segment of interest, key point extraction, and preprocessing into normalized model-ready arrays, rather than pose extraction alone. The output was saved in JSONL format with one frame per line, retaining both the seven selected joints and the full 308-keypoint representation for potential future analysis. All processing reported in this study was performed offline on stored recordings. We did not evaluate the pipeline in a streaming configuration.

We applied per-frame normalization to remove camera-specific variation in scale and position. First, the pose was centered on the midpoint between the two shoulders. This centering removes translation effects that depend on the participant position relative to the camera. Second, the pose was scaled by the shoulder width measured in pixels. This scaling normalizes for participant size and for camera distance. The final normalized pose represents joint positions in shoulder-width units relative to the shoulder midpoint.

Frames were marked as missing if either shoulder had detection confidence below 0.3 or if the computed shoulder width fell below 10^−6^ in normalized image coordinates. The latter condition guards against numerical instability when the shoulders are too close together to define a reliable scale. Marking such frames as missing rather than imputing values prevents corruption of downstream statistics by unreliable detections.

Pose sequences were segmented into temporal windows for downstream modeling. Each window contained 30 consecutive frames, corresponding to 1 s of recording at 30 frames per second. Windows overlapped with a hop size of 15 frames, corresponding to 0.5 s. Windows containing more than 50% missing frames were discarded. Missing frames in retained windows were imputed with zeros. The resulting window representation had shape (*W*, 30, 14), where *W* denotes the number of windows in the dyad and 14 represents seven joints multiplied by two coordinates.

We computed per-window velocity through frame-level differencing. The velocity component had the same shape as the position component. We concatenated position and velocity along the feature dimension to produce a final window representation of shape (*W*, 30, 28). Concatenating velocity provides the model with both positional and dynamic information without requiring it to learn temporal derivatives. We applied per-feature z-score normalization within each dyad to remove individual baseline differences. Outlier values were clipped to the range of negative five to positive five standard deviations to prevent rare extreme values from dominating the loss.

### 2.6 DXSP architecture

Figure 2 presents an overview of the DXSP architecture. Windows from both participants pass through a shared window encoder. Two cross-attention modules model each participant against the other, and their outputs are concatenated into a dyad-level representation. The concordance gate reads the encoder outputs, so interpersonal synchrony is measured before cross-attention mixes the two streams. An attention head scores every window, and a hard top-*k* mask retains exactly *k* = 3 positions. The attention distribution and the gate are combined element wise and renormalized, then used to pool the dyad representation for classification. The model returns two outputs, a predicted outcome and the *k* selected positions.

**Figure 2:**
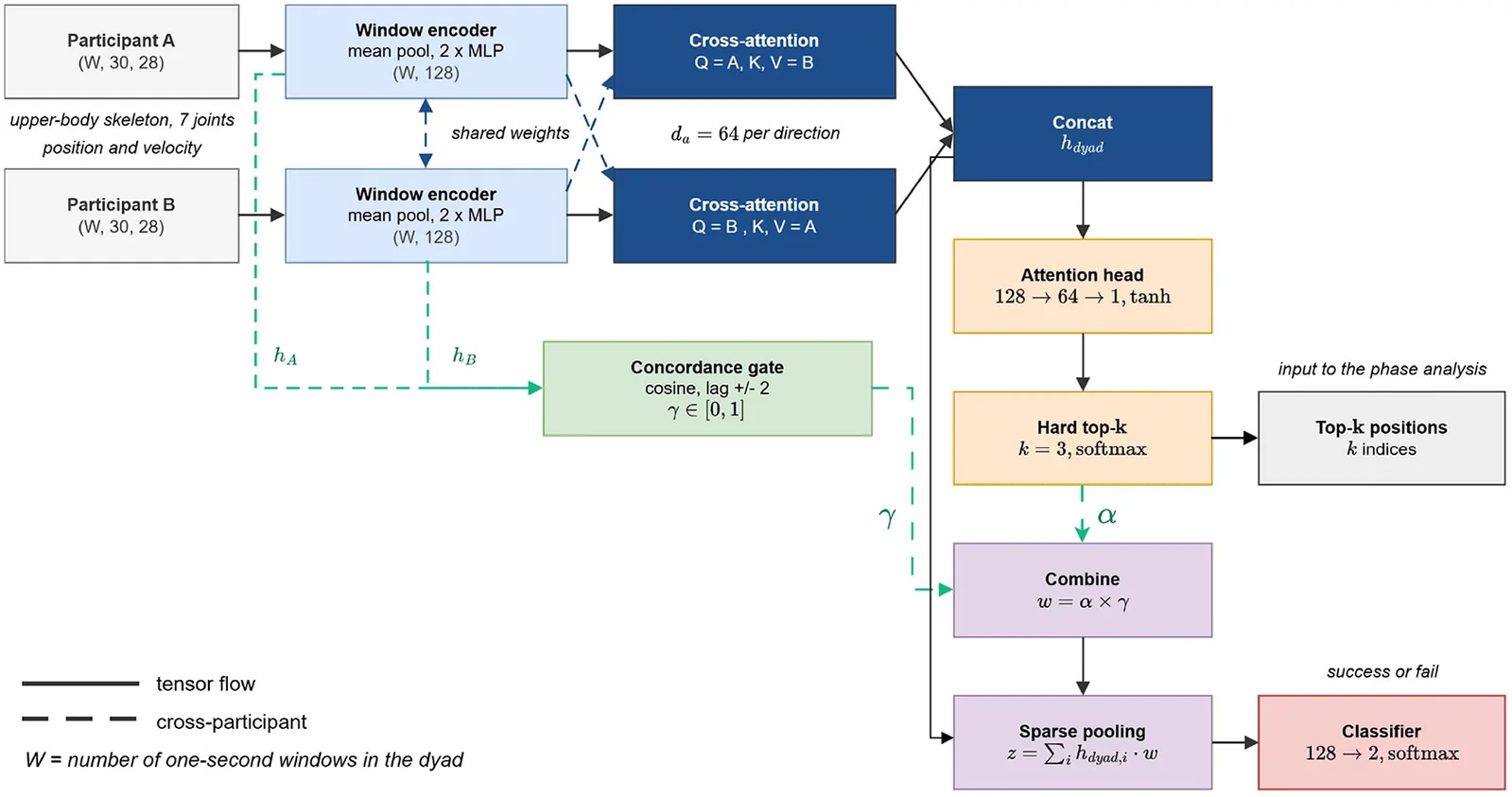
Overview of the DXSP architecture. Windows from both participants pass through a shared encoder, cross-attention, a synchrony-based concordance gate, and a hard top-*k* selection with *k* = 3, before sparse pooling and classification. The model returns both a predicted outcome and the k selected window positions.

#### 2.6.1 Window encoder

Each input window has shape (30, 28) corresponding to 30 frames of 28-dimensional position-and-velocity features. The encoder first applies mean pooling across the temporal dimension to produce a 28-dimensional vector. This discards the ordering and shape of movement within the window, although motion is not lost entirely, since velocity is computed by frame-level differencing before pooling and the pooled vector therefore carries the mean rate of change alongside the mean position. Structure above 1 s is preserved by the window sequence, which advances at the hop size of 0.5 s and is the level at which decisive moments are localized. The 1-s window was fixed a priori rather than tuned. The rhetorical phases analyzed in this study span tens of seconds, so a 1-s window is far finer than the structure the analysis targets while remaining long enough to contain a complete gesture stroke. We did not sweep the window size, and an adaptive window remains untested. The pooled vector is then projected through a two-layer feedforward network with hidden dimension *d*_*h*_ = 128. Each layer consists of a linear projection, layer normalization, ReLU activation, and the first layer adds dropout with rate 0.2. The output is a window embedding of dimension 128. The same encoder weights are applied to both participants. This weight sharing reduces parameter count and reflects the symmetric nature of the dyadic interaction.

#### 2.6.2 Cross-attention

To model the dependency of one participant gesture context on the other, we apply two parallel cross-attention modules. The first module uses participant A embeddings as queries and participant B embeddings as keys and values. The second module reverses these roles. Each cross-attention module uses scaled dot-product attention with attention dimension *d*_*a*_ = 64. The output of the first module is a context-aware representation for participant A that reflects how participant B gestures shape the interpretation of participant A gestures. The output of the second module is the analogous representation for participant B. We concatenate these two outputs along the feature dimension to produce a dyad-level representation.

#### 2.6.3 Concordance gate

To weight candidate moments by interpersonal synchrony, we compute a concordance score for each window. We use cosine similarity between the two participant embeddings in the latent encoding space. Simple frame-aligned cosine similarity is too strict in practice because the two participants may exhibit synchronized gestures with small temporal lags. To address this, we allow a lag tolerance of two windows, corresponding to 1 s around the target window. The lag-tolerant concordance for each window is the maximum cosine similarity over lags in the range from negative two to positive two. We rescale the cosine similarity from the range minus one to plus one into the range zero to one for downstream multiplication with the attention distribution. Cosine similarity is used rather than a distance measure because it compares direction independently of magnitude, so a dyad in which one participant gestures more expansively than the other is not penalized. The lag tolerance reflects that interpersonal synchrony is rarely frame-aligned, and taking the maximum over lags captures both leading and following relations within a 1-s neighborhood.

#### 2.6.4 Hard top-*K* attention

To enforce structural sparsity, we use a hard top-*k* attention mechanism with *k* = 3. We compute attention logits for each window using a two-layer attention head applied to the dyad-level representation. The head consists of a linear projection to dimension 64, hyperbolic tangent activation, and a final linear projection to scalar logits. We then construct a binary mask that retains only the *k* largest logits. We add a large negative constant to logits at non-selected positions before applying softmax. The resulting attention distribution has support on exactly *k* positions. All other positions receive zero weight. The choice of *k* fixes how many windows receive attention mass but places no constraint on their temporal positions.

This hard top-*k* mechanism distinguishes DXSP from prior soft-attention architectures. Soft-attention models rely on entropy or L1 regularization to encourage sparsity. These soft methods do not guarantee that attention concentrates on a small number of positions. Empirical evaluation in our preliminary development cycles showed that soft regularization produced attention distributions with entropy close to the uniform maximum, even with strong regularization weights. The hard top-*k* mechanism guarantees by construction that *k* of *W* windows receive 100% of the attention mass.

#### 2.6.5 Pooled representation and classifier

We combine the attention distribution and the concordance gate through element-wise multiplication. The resulting combined weight is renormalized so that the weights sum to one. The pooled dyad representation is computed as the weighted sum of the dyad-level features weighted by the combined weight. We then apply a linear classification head to the pooled representation to produce binary logits for the two persuasion outcomes. We apply softmax to obtain class probabilities.

The element-wise multiplication of attention and concordance implements a logical conjunction in soft form. A window contributes to the pooled representation only if both the attention head considers it informative and the participants are synchronized at that window. This conjunction encodes the hypothesis that decisive persuasive moments may require both task relevance and interpersonal coordination.

### 2.7 Training procedure

We trained DXSP using leave-one-dyad-out cross-validation. For each of the 46 dyads, we held out that dyad as the test set and trained on the remaining 45 dyads. This protocol ensures that no participant appears in both training and test sets. The protocol also provides a single prediction for every dyad in the dataset. The total number of model training runs was therefore 46.

We used the AdamW optimizer with learning rate 1 × 10^−3^ and weight decay 1 × 10^−4^. The loss function combined cross-entropy on the binary classification target with a small total variation regularizer on the attention distribution. The total variation term penalizes large differences between consecutive attention values. The regularization weight was set to 0.05. This light regularization encourages smooth temporal structure without forcing attention into specific positions. We applied gradient clipping at a norm of 5.0 to prevent training instability from rare extreme gradient magnitudes.

We trained each fold for 30 epochs. Each epoch passed through the training dyads in randomized order with stochastic gradient updates one dyad at a time. We did not use batch updates because of the variable number of windows per dyad. Random seeds were fixed across all stochastic operations to ensure reproducibility. The training procedure was implemented in PyTorch version 2.0 on Python 3.10. Training time per fold was approximately 14 s on the workstation graphics card, yielding a total cross-validation time of approximately 10 min.

### 2.8 Statistical analysis

We applied seven classes of statistical tests to the model outputs and the derived peak positions. We describe each class below.

#### 2.8.1 Mode-fitting analysis

We fit Gaussian Mixture Models with 1 through 5 components on the pooled top-*k* peak positions. Each model was fit with 10 initializations to mitigate local optima. Model selection used the Bayesian Information Criterion (Schwarz, 1978). We followed the convention established by Kass and Raftery (1995) that a difference in BIC greater than 10 constitutes decisive evidence for the more complex model. Smaller BIC differences indicate weaker evidence. The selected model serves as the basis for all subsequent mode-related analyses. Because these positions are selected by the model rather than specified in advance, we calibrated the comparison against a matched null in which *k* windows were drawn uniformly at random from each dyad, using *B* = 1, 000 replicates. We repeated this for *k* from 1 to 10.

#### 2.8.2 Bootstrap mode stability

We assessed the stability of mode positions by resampling the pooled peak positions with replacement *B* = 1, 000 times. For each resample we refit the 3-component Gaussian Mixture Model and recorded the sorted mode centers. We reported the 95 percentile confidence intervals as the lower 2.5 and upper 97.5 percentiles of the bootstrap distribution. Narrow and non-overlapping confidence intervals indicate stable mode positions that are unlikely to be driven by sampling variability.

#### 2.8.3 Chi-square test of independence

We tested association between persuasion outcome and phase using the standard Pearson chi-square test on the 2 by 3 contingency table of outcome times phase counts. We reported the test statistic, degrees of freedom, and asymptotic p-value. The effect size was reported as Cramér *V* with conventional thresholds from Cohen (1988). Cramér *V* values of 0.10, 0.30, and 0.50 indicate small, medium, and large effects respectively. The chi-square test assumes independent observations. Because each dyad contributes *k* = 3 peaks, the 138 observations are not strictly independent. We therefore complement the asymptotic chi-square with the permutation test described below.

#### 2.8.4 Permutation test for non-independence

We performed a permutation test that preserves the within-dyad clustering structure. We shuffled dyad-level outcome labels *P* = 10000 times. For each permutation we recomputed the chi-square statistic on the resulting contingency table. The empirical p-value was the proportion of permutations yielding a test statistic greater than or equal to the observed value. This test does not rely on asymptotic distributional assumptions and properly accounts for the non-independence of multiple peaks per dyad.

#### 2.8.5 Dyad-level Mann–Whitney *U* tests

As a complementary analysis using strictly independent observations, we computed each dyad fraction of peaks in each phase. We compared distributions between outcome groups using two-sided Mann–Whitney *U* tests. We applied Benjamini-Hochberg false discovery rate correction across the three phase comparisons. The effect size was reported as rank-biserial correlation. This test treats each dyad as a single observation and therefore satisfies the independence assumption.

#### 2.8.6 Bootstrap confidence interval for effect size

We computed a percentile bootstrap 95% confidence interval for Cramér *V* over *B* = 1000 dyad-level resamples. This interval characterizes the precision of the effect size estimate independent of significance testing. A lower bound exceeding the threshold for small effects of 0.10 indicates that the association is substantively reliable beyond statistical significance.

#### 2.8.7 Joint-level tests

For each dyad and each of the seven joints we computed the mean velocity magnitude at the top-*k* windows and the mean over all windows and took the difference. The unit of analysis is therefore the dyad, which gives 46 independent observations per joint. We tested each joint difference against zero using a two-sided Wilcoxon signed-rank test. We applied Holm-Bonferroni correction across the seven joints. The effect size was reported as the rank-biserial correlation, with a percentile bootstrap 95% confidence interval over B = 1000 dyad-level resamples.

All analyses were performed in Python 3.10 using SciPy version 1.10, statsmodels version 0.14, and scikit-learn version 1.3. Random seeds were fixed across all stochastic operations to ensure reproducibility.

## 3 Results

### 3.1 Temporal distribution of dyadic attention peaks

Following leave-one-dyad-out cross-validation training, we extracted top-*k* attention positions from each of the 46 dyads using *k* = 3. This procedure yielded a total of *N* = 138 normalized peak positions in the interval from 0 to 1 for subsequent analysis.

*Distribution across rhetorical phases*. We partitioned the interaction timeline into three equal phases motivated by Rhetorical Structure Theory (Mann and Thompson, 1988). The opening phase covers the interval from 0 to 0.33. The middle phase covers from 0.33 to 0.67. The closing phase covers from 0.67 to 1.0. Top-*k* peaks were nearly uniformly distributed across these phases. The opening phase contained 46 of 138 peaks, corresponding to 33.3%. The middle phase contained 45 peaks, corresponding to 32.6%. The closing phase contained 47 peaks, corresponding to 34.1%. All three phases received attention across dyads. The pooled distribution is therefore close to uniform, and it gives no evidence that decisive moments concentrate in any single phase.

*Mode-fitting analysis*. To formally test whether the peak distribution exhibits multi-modal structure, we fit Gaussian Mixture Models with 1, 2, 3, 4, and 5 components. We selected the model minimizing the Bayesian Information Criterion. The BIC values were 68.85, 58.36, 52.32, 65.51, and 75.49 for 1, 2, 3, 4, and 5 components respectively. The 3-component model achieved the lowest BIC. The difference relative to the 1-component model was ΔBIC of 16.53. This difference exceeds the conventional threshold of 10 given by Kass and Raftery (1995) but that threshold assumes a comparison specified independently of the data, which is not the case here. The 3-mode centers were located at normalized positions 0.090, 0.439, and 0.815. These centers are nearly equally spaced and correspond closely to the opening, middle, and closing phase centers.

*Bootstrap stability of mode positions*. To assess the stability of the inferred modes, we resampled the 138 peak positions with replacement *B* = 1, 000 times. We refit the 3-component Gaussian Mixture Model for each resample. The resulting 95% confidence intervals for mode centers were tight and non-overlapping. Mode 1 was located at 0.090 with 95% confidence interval from 0.063 to 0.170. Mode 2 was located at 0.439 with 95% confidence interval from 0.343 to 0.552. Mode 3 was located at 0.815 with 95% confidence interval from 0.733 to 0.886. The narrow widths and clear separation of these intervals indicate that the fitted mode positions are stable under resampling of the same peaks. Stability under resampling does not establish that the three-mode fit is preferable to a simpler description. That question is addressed by the calibration below.

*Null calibration*. Because the peak positions are selected by the model rather than specified in advance, we calibrated the mixture comparison against a matched null in which *k* windows were drawn uniformly at random from each dyad. Across 1,000 replicates, the null distribution of ΔBIC had a mean of 8.27 and a 95th percentile of 26.01, and 30.5% of replicates also selected three components. The observed ΔBIC of 16.53 corresponds to an empirical p of 0.210. Selecting the *k* highest-energy windows without any learned attention gave a larger ΔBIC of 18.70. Repeating the analysis for *k* from 1 to 10 gave no value of *k* exceeding its own null at the 0.05 level, and the selected number of components was not stable across *k*. We therefore report the mode positions as descriptive rather than as evidence for three-phase organization.

Across all 46 dyads, DXSP-identified decisive moments were distributed across the full interaction. A three-component mixture places components near the opening, middle, and closing phase centers, but this structure is not distinguishable from what random selection under the same sparsity constraint produces. This finding is summarized in Figure 3.

**Figure 3:**
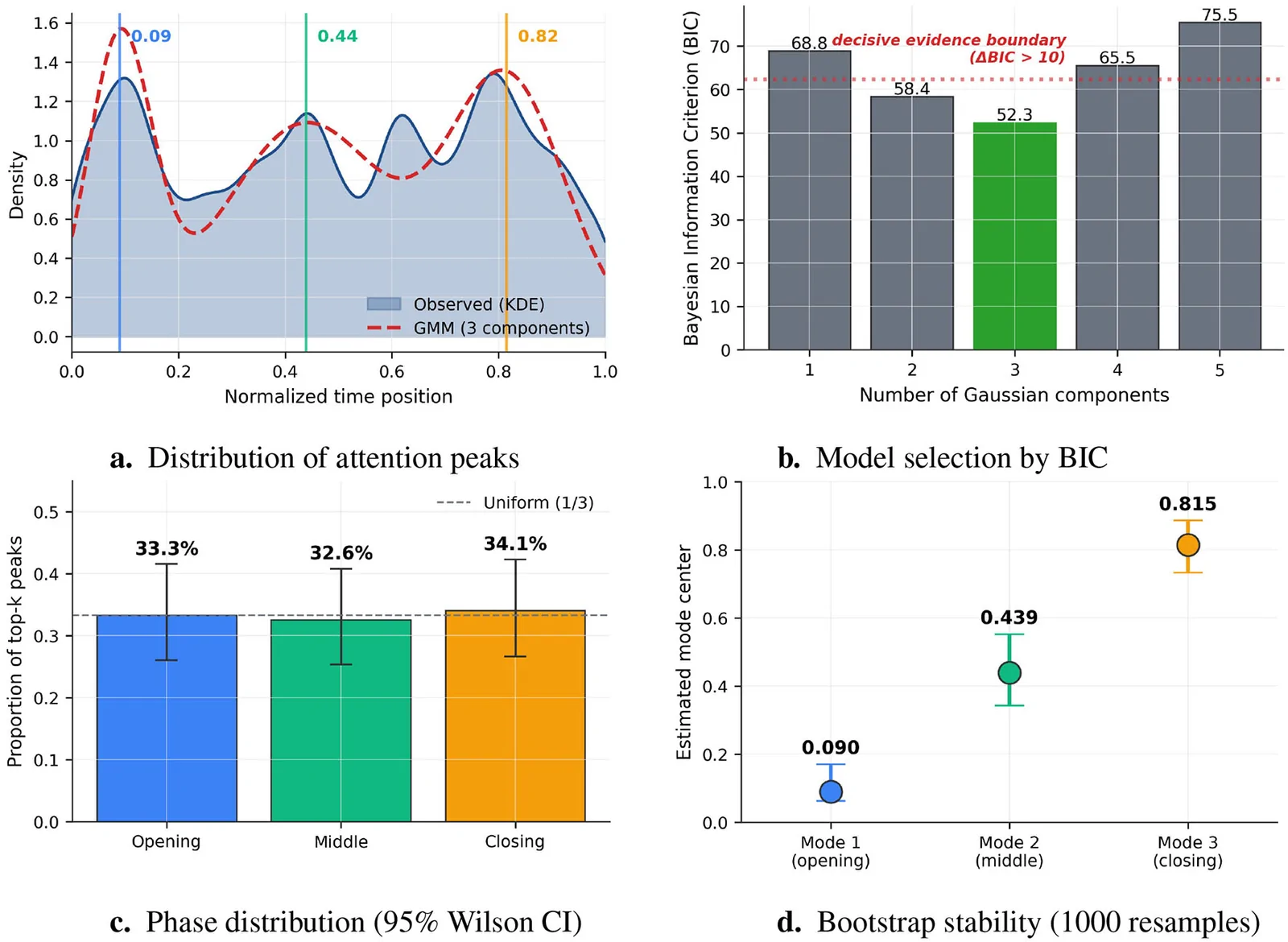
Temporal distribution of attention peaks. **(a)** Kernel density estimate (shaded blue) and 3-component Gaussian Mixture Model fit (dashed red) over the 138 normalized peak positions, with mode centers marked. **(b)** Bayesian Information Criterion (BIC) values for Gaussian Mixture Models with 1 to 5 components. The 3-component model has the lowest BIC. The dotted line marks the conventional threshold of 10 BIC units and is shown for reference only, since Section 3.1 reports that this threshold does not apply to model-selected positions. **(c)** Phase distribution with 95% Wilson confidence intervals. The dashed gray line indicates the uniform expectation of one third. **(d)** Bootstrap stability of mode centers across 1000 resamples with 95 percentile confidence intervals.

### 3.2 Phase distribution of decisive moments by persuasion outcome

Independently of the mixture analysis, we asked whether the relative concentration of decisive moments across phases differs by persuasion outcome. Dyads were dichotomized into successful and failed groups using the mean rated-persuasion score across both participants. Successful dyads had mean score greater than or equal to 4 (*n* = 19). Failed dyads had mean score less than or equal to 3 (*n* = 27). No dyads fell into the ambiguous range with mean score between 3 and 4.

*Per-phase peak counts*. Aggregating top-*k* peaks by outcome and phase produced the counts summarized in Figure 4, which plots them as within-group proportions. Successful dyads contributed 28 opening peaks, 11 middle peaks, and 18 closing peaks. Failed dyads contributed 18 opening peaks, 34 middle peaks, and 29 closing peaks. The proportional patterns differ between groups. Successful dyads concentrated 49.1% of their peaks in the opening phase. Failed dyads concentrated 42.0% of their peaks in the middle phase.

**Figure 4:**
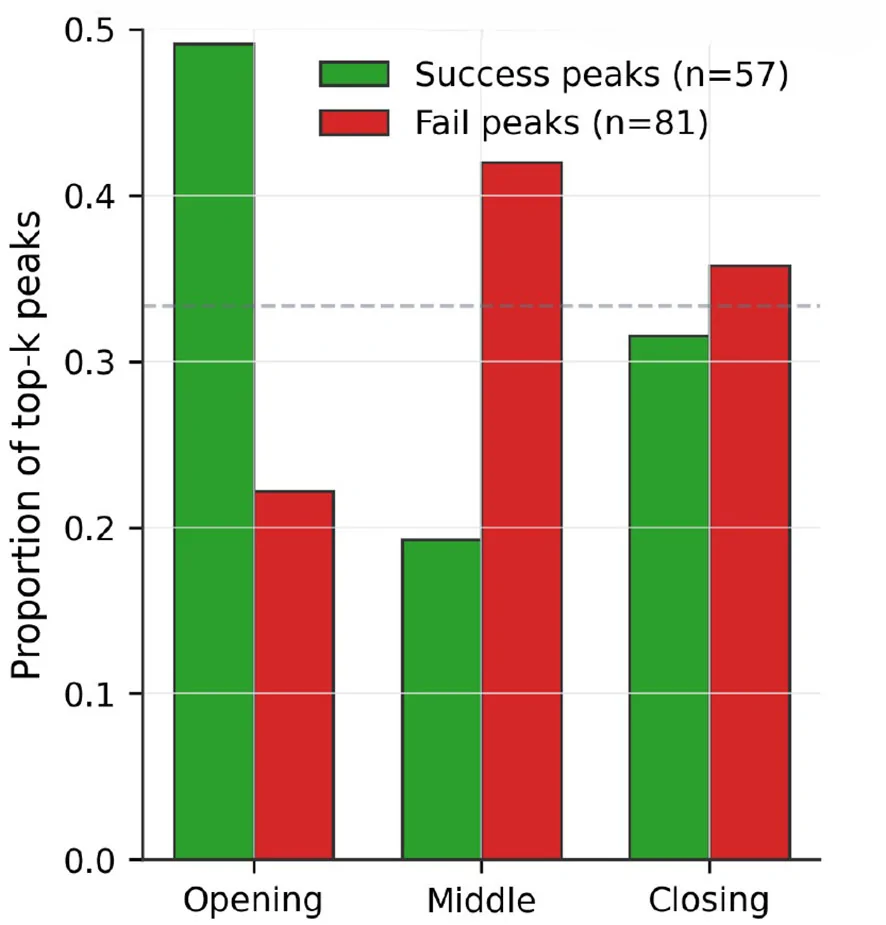
Phase distribution of top-*k* peaks by outcome. Green bars show successful dyads. Red bars show failed dyads. The dashed gray line indicates the uniform expectation of one third.

*Aggregate chi-square test:* A standard Pearson chi-square test of independence on the 2 by 3 contingency table yielded χ^2^ = 12.72 with 2 degrees of freedom. The asymptotic p-value was 0.0017. The effect size measured by Cramér *V* was 0.304. According to conventions established by Cohen (1988), this value represents a medium effect.

*Permutation test for non-independence:* Because each dyad contributes three peaks, the 138 observations are not statistically independent. This non-independence may inflate the asymptotic test statistic. We therefore performed a permutation test by shuffling dyad-level outcome labels *P* = 10000 times. For each permutation we recomputed the chi-square statistic on the resulting contingency table. This procedure preserves the within-dyad clustering structure. The empirical p-value was 0.020. The permutation *p*-value is larger than the asymptotic *p*-value, as expected when within-dyad clustering is taken into account. The result remained below 0.05.

*Dyad-level Mann–Whitney U tests:* As a complementary analysis using strictly independent observations, we computed the fraction of peaks falling in each phase for each individual dyad. We then compared the distributions between outcome groups using two-sided Mann–Whitney *U* tests. We applied the Benjamini-Hochberg false discovery rate correction across the three phase comparisons. The opening phase showed a significant difference. The successful mean fraction was 0.491. The failed mean fraction was 0.222. The test statistic was *U* = 372.0 with FDR-adjusted p of 0.020. The rank-biserial correlation was −0.450, representing a medium-to-large effect. The middle phase showed a non-significant trend. The successful mean fraction was 0.193. The failed mean fraction was 0.420. The test statistic was *U* = 177.5 with FDR-adjusted p of 0.094. The rank-biserial correlation was +0.308. The closing phase showed no detectable difference. The successful mean fraction was 0.316. The failed mean fraction was 0.358. The test statistic was *U* = 242.5 with FDR-adjusted *p* of 0.751. The rank-biserial correlation was +0.055.

*Bootstrap confidence interval for effect size:* To characterize the precision of the observed association, we computed a bootstrap 95% confidence interval for Cramér *V* over *B* = 1000 dyad-level resamples. The bootstrap estimate yielded *V* of 0.304 with 95% confidence interval from 0.141 to 0.488. The lower bound exceeds the threshold for small effects of 0.10. Successful persuasion in our sample is associated with earlier concentration of decisive gesture moments. Failed persuasion is associated with mid-interaction concentration. Figure 5 visualizes this asymmetry at the level of individual dyads, with each dyad represented as a single point in each panel.

**Figure 5:**
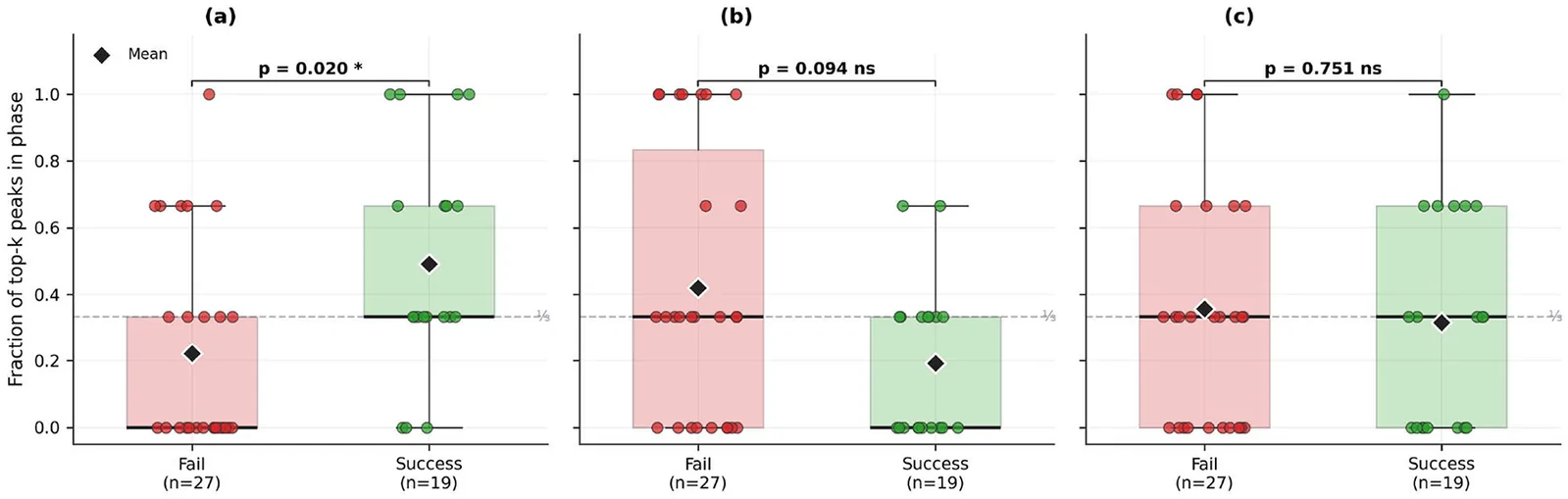
Dyad-level phase distribution by persuasion outcome (*n* = 46 dyads). Each panel shows the fraction of top-*k* peaks falling in one phase, separately for successful and failed dyads. Box plots show the interquartile range with median lines. Individual dyads are shown as scatter points with horizontal jitter for clarity. Diamond markers indicate group means. Significance brackets show FDR-corrected p-values from Mann–Whitney *U* tests. The dashed gray line indicates the uniform expectation of one third. **(a)** Opening phase. **(b)** Middle phase. **(c)** Closing phase.

### 3.3 Joint-level signatures of decisive moments

To support the interpretability claim of DXSP and to provide design-relevant information for downstream persuasive AI systems, we decomposed the gesture energy at decisive moments by anatomical joint.

*Aggregate per-joint contributions:* For each window, we computed the velocity magnitude per joint by combining contributions from participants A and B. We then compared the mean energy at top-*k* windows to the mean across all windows for each of the seven Sapiens Goliath upper-body joints. Table 1 reports the per-joint energy comparison ranked by top-*k* mean magnitude. Figure 6b shows the same comparison graphically, ranking the seven joints by mean magnitude at top-*k* windows against the mean across all windows.

**Table 1:** Per-joint velocity magnitude at top-*k* decisive windows compared to all windows.

Joint	Top-*k* mean	All-windows mean	Difference
Nose	1.835	1.749	+0.086
Left elbow	1.476	1.460	+0.016
Right elbow	1.399	1.421	−0.021
Left wrist	1.387	1.317	+0.069
Right shoulder	1.307	1.300	+0.007
Left shoulder	1.307	1.300	+0.007
Right wrist	1.260	1.324	−0.064

**Figure 6:**
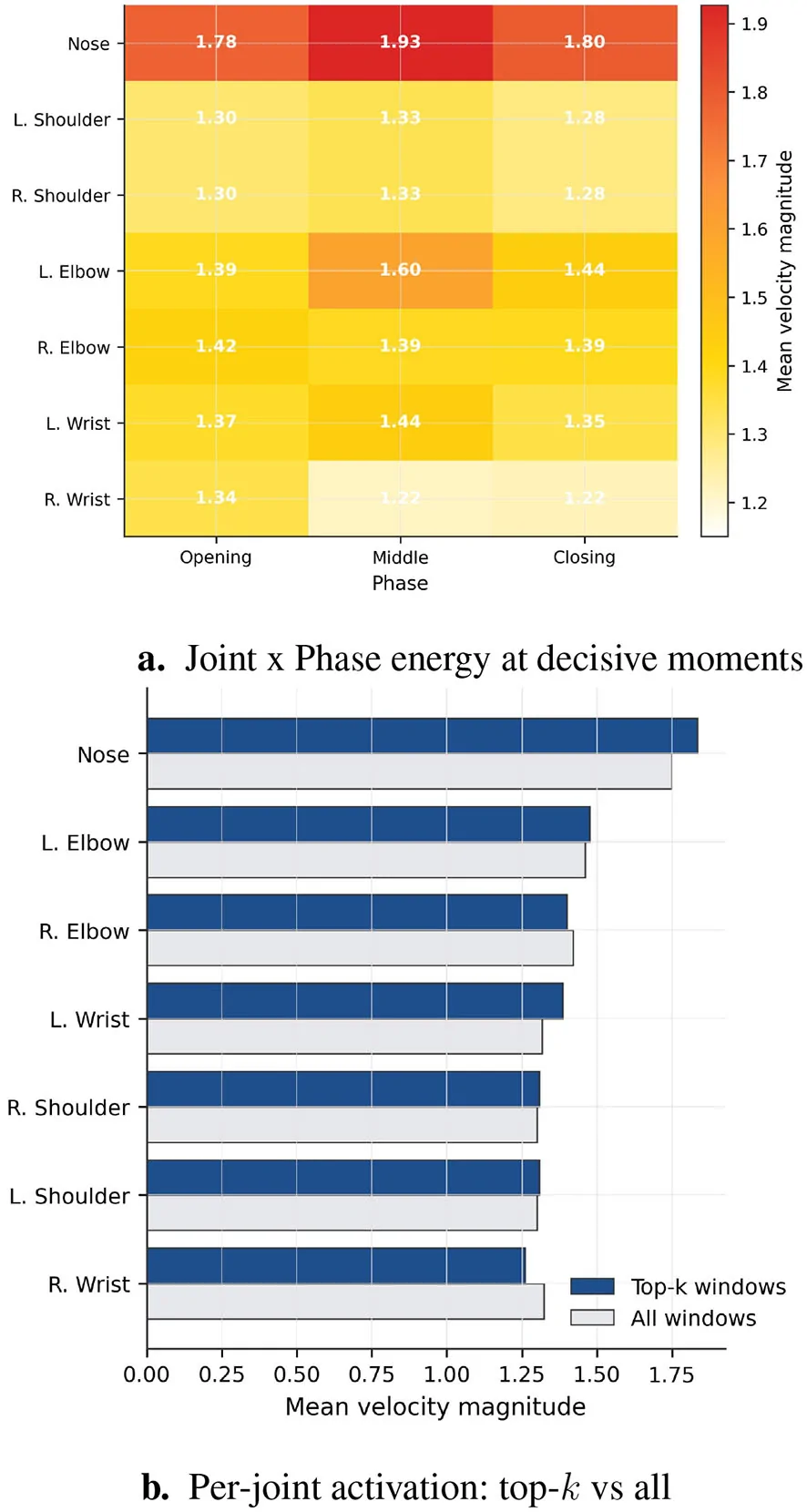
Joint-level interpretability of decisive moments. **(a)** Heat map showing mean velocity magnitude for each of the seven joints across the three phases. The color scale is shared across cells. The nose joint shows the highest activation across all phases, with peak activity in the middle phase. **(b)** Per-joint activation ranked by mean magnitude at top-*k* windows compared to mean across all windows. The horizontal bars show that the nose joint has the largest positive difference between top-*k* and baseline windows.

The nose joint exhibited the largest positive difference, with top-*k* mean of 1.835 and all-windows mean of 1.749, yielding a difference of +0.086. The left wrist joint showed the second largest positive elevation, with top-*k* mean of 1.387 and all-windows mean of 1.317, yielding a difference of +0.069. The left elbow joint showed a smaller positive difference of +0.016. Both shoulder joints showed negligible differences of +0.007. The right elbow joint showed a small negative difference of −0.021. The right wrist joint showed a negative difference of −0.064. Tested across dyads with Wilcoxon signed-rank tests and Holm-Bonferroni correction over the seven joints, none of these differences reached significance. The nose difference gave a raw *p* of 0.435 and a Holm-adjusted *p* of 1.000, with a bootstrap 95% confidence interval from –0.067 to 0.246 that includes zero. The smallest raw *p* across the seven joints was 0.064 for the right wrist, which became 0.451 after correction. The Friedman test across the seven paired differences gave chi-square of 4.873 with 6 degrees of freedom and *p* of 0.560, with Kendall W of 0.018, so the ranking does not indicate that any joint is specifically elevated at decisive windows. We therefore report this ranking as exploratory. The two shoulders are identical by construction, since centering on the shoulder midpoint and scaling by shoulder width makes them exact mirror images. The shoulder joints serve as anatomical references rather than expressive endpoints, as expected for upper-body skeletal anchoring.

*Phase by joint structure:* Decomposing energy by both phase and joint shows the following descriptive patterns across the three rhetorical phases (Figure 6a). The nose joint exhibits the highest energy across all three phases. Its peak activation occurs during the middle phase with mean velocity magnitude of 1.927, compared to 1.782 in the opening phase and 1.798 in the closing phase. The left elbow joint also displays elevated middle-phase activity with mean magnitude of 1.601, consistent with sustained gestural emphasis during mid-argument exchanges. Wrist asymmetry is notable. The left wrist activity is highest in the middle phase at 1.442. The right wrist activity peaks in the opening phase at 1.341 and declines thereafter.

The nose joint showed the largest mean elevation at decisive moments, but this difference did not survive correction across the seven joints, so we do not treat head orientation as an established signal of persuasive engagement. Head movement and gaze direction have been reported as markers of engagement in prior work (Bohus and Horvitz, 2010) and testing whether that holds for gesture-only decisive moments would require a larger sample than ours. The pattern is also relevant to recent work on attention-based dyadic engagement modeling (Li et al., 2023; Funk et al., 2024).

### 3.4 Outcome prediction performance

While the temporal structure of decisive moments differentiates persuasion outcomes as shown in Section 3.2, we also evaluated whether the predicted probabilities from DXSP can directly classify individual dyads as successful or failed.

Under leave-one-dyad-out cross-validation, DXSP achieved an area under the receiver operating characteristic curve (AUC) of 0.536. The classification accuracy was 56.5%. These metrics are shown in Figures 7a, b. For comparison, a trivial baseline that always predicts the majority class would achieve 27 of 46 correct predictions. This baseline yields an accuracy of 58.7%. The confusion matrix indicates that the DXSP model is biased toward predicting the fail class. The specificity for the fail class is 74.1%. The sensitivity for the success class is only 31.6%.

**Figure 7:**
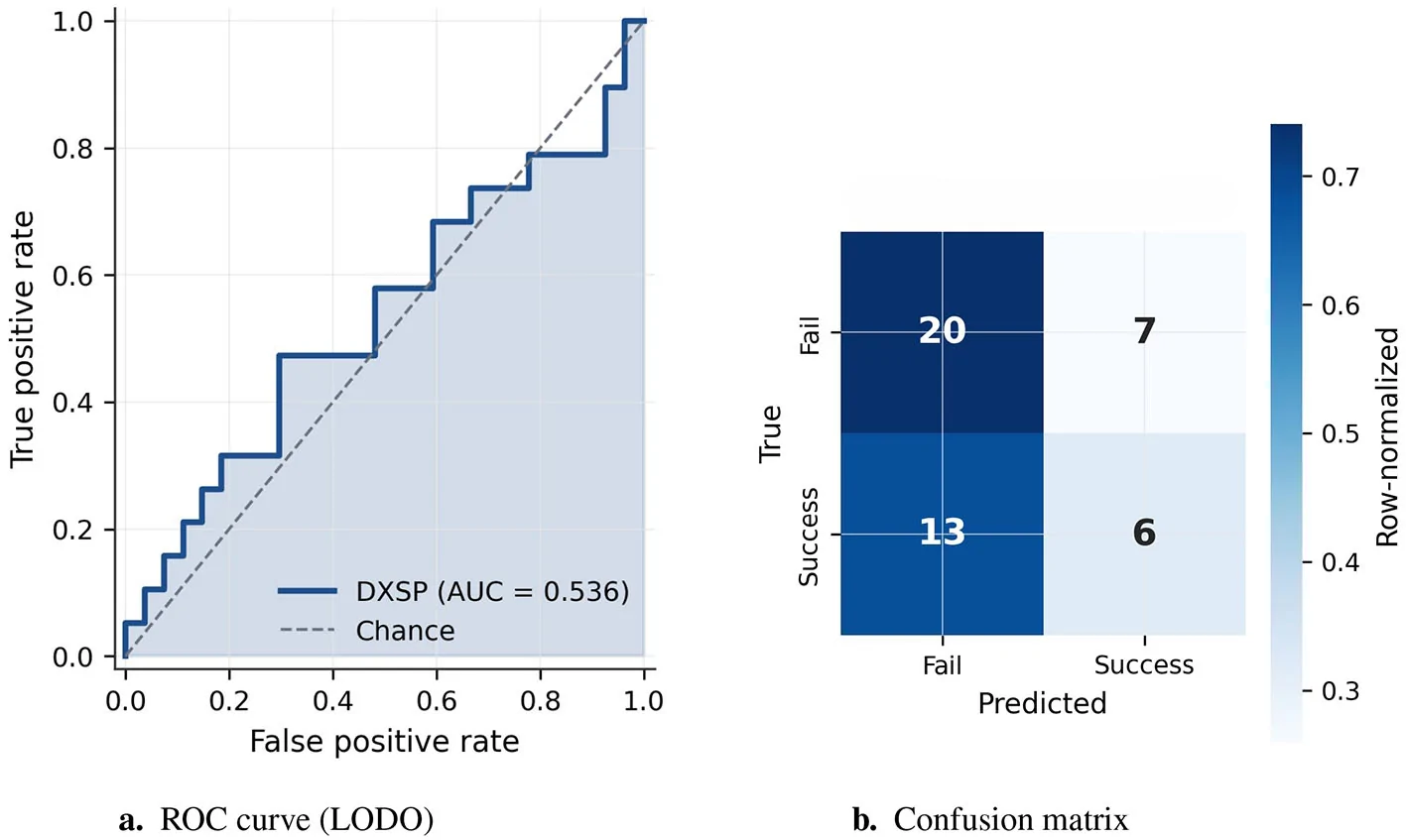
Outcome prediction analysis. **(a)** Receiver operating characteristic curve for DXSP under leave-one-dyad-out cross-validation. The dashed diagonal indicates chance performance. **(b)** Confusion matrix annotated with raw counts. Row-normalized colors indicate prediction proportions.

Table 2 reports leave-one-dyad-out performance for DXSP against three recent skeleton-based architectures and a classical aggregate-feature baseline. All models were trained under the identical protocol, optimizer, epoch budget, and seeds, so the comparison isolates architecture rather than training recipe. No model separates persuasion outcome above chance. No bootstrap interval in the table lies entirely above 0.5 and the highest AUC in the table is 0.536. We interpret this as showing that persuasion outcome is not predictable from gesture alone at this sample size, rather than as a ranking among architectures. DXSP produces exactly *k* positions per dyad by construction. The baselines pool over all windows and produce no decisive-moment positions at all. The contribution of the hard top-*k* mechanism is therefore localization rather than classification accuracy.

**Table 2:** Comparison against state-of-the-art approaches.

Model	AUC	95% CI
BlockGCN (Zhou et al., 2024)	0.470	[0.299, 0.643]
SkateFormer (Do and Kim, 2025)	0.327	[0.170, 0.494]
ProtoGCN (Liu et al., 2025)	0.442	[0.268, 0.609]
Random forest (aggregate features)	0.314	[0.163, 0.483]
**DXSP**	**0.536**	**[0.362, 0.715]**

Bold indicates the model proposed in this study.

The baselines are reimplementations adapted to this corpus rather than original code released by the authors and they do not use published checkpoints. Two adaptations were unavoidable. This corpus provides a seven-joint upper-body graph rather than the 25-joint NTU graph, and a dyad is a variable-length sequence of 1-s windows rather than a trimmed action clip. Reported numbers should therefore be read as performance under this protocol, not as the published performance of these methods.

This modest prediction performance contrasts with the temporal-structural finding in Section 3.2. We interpret this contrast as evidence of a positional content distinction. DXSP appears to capture when persuasion-relevant moments occur, corresponding to positional structure. The content at those moments, as captured by latent representations of synchrony and energy, does not directly determine the outcome at this sample size. We return to the theoretical and practical implications of this distinction in Section 4.

### 3.5 Per-dyad case studies

To provide concrete illustrations of the patterns identified above, Figure 8 visualizes the attention timelines of four dyads. Each panel overlays three trajectories on the normalized time axis. The first trajectory shows concordance, which measures synchronization between participants. The second trajectory shows total energy, which combines velocity magnitudes across both participants. The third visual element consists of top-*k* attention bars indicating the decisive moment positions. Phase boundaries are indicated by background shading.

**Figure 8:**
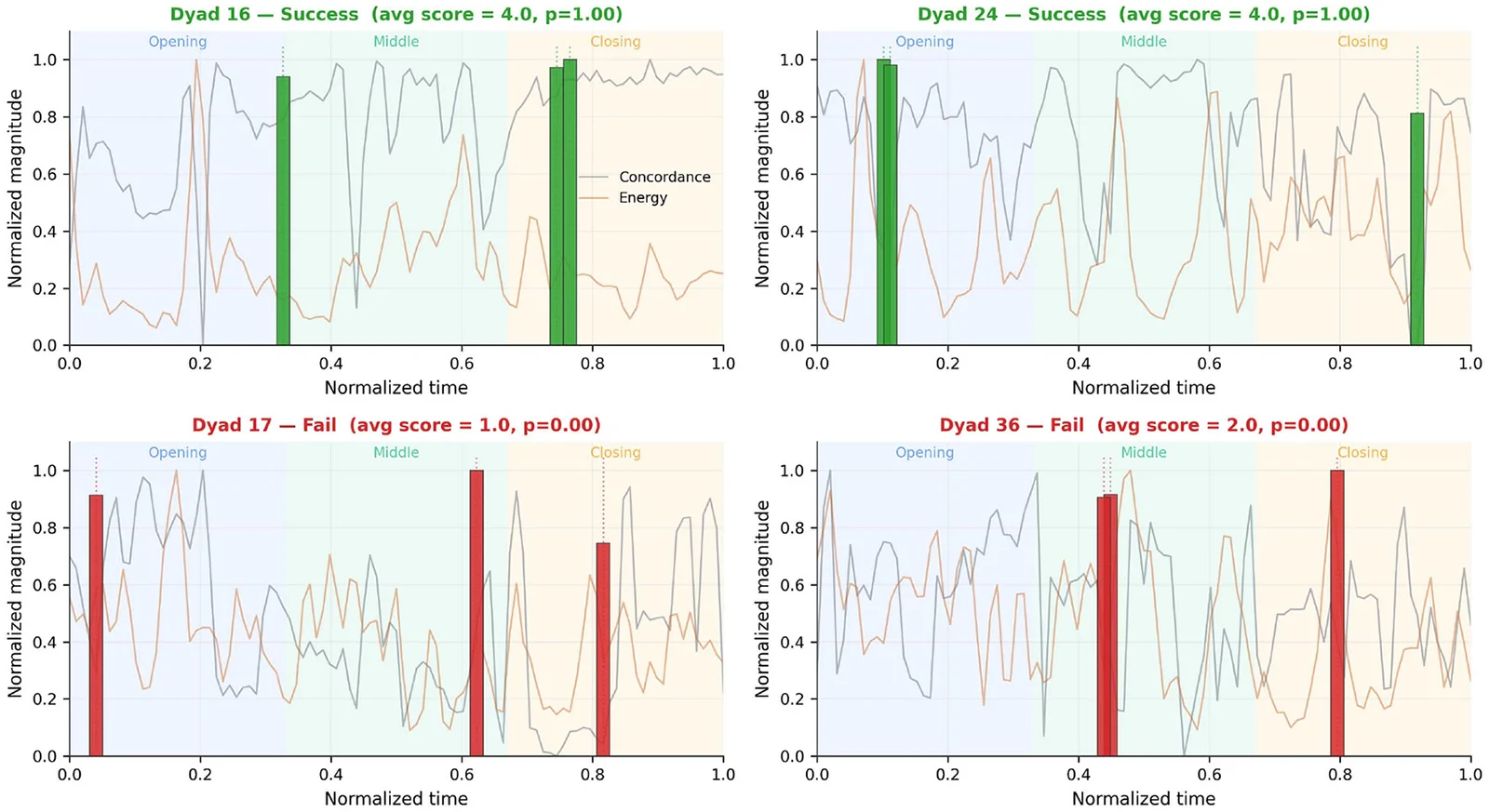
Per-dyad attention case studies. The four panels show two successful dyads [**(Top row)**, green attention bars] and two failed dyads [**(Bottom row)**, red attention bars], selected by highest predicted success and failure probability respectively. Each panel shows concordance trajectory in gray and energy trajectory in orange, both normalized to the unit interval. Vertical bars indicate top-*k* decisive moment positions with height proportional to attention weight. Background shading indicates the three rhetorical phases.

The four panels in Figure 8 were selected by highest predicted success probability for the top row and highest predicted failure probability for the bottom row. This criterion is based on model confidence and is unrelated to phase distribution, so these dyads were not chosen to exemplify the aggregate pattern and should not be read as evidence for it. Peaks appear near phase boundaries and in the closing phase in both groups. This is consistent with the dyad-level distributions in Figure 5 where the outcome groups overlap substantially despite differing in mean opening-phase fraction. Peaks in these panels are sometimes accompanied by elevated concordance, in both successful and failed dyads. The four panels are therefore presented as illustrations of dyad-level variability. The aggregate claim rests on the tests in Section 3.2 rather than on these examples.

## 4 Discussion

### 4.1 Mixture evidence and its calibration

A three-component mixture fits the selected positions. That fit alone could be read as evidence for three temporal clusters of decisive gesture moments. The null calibration does not support that finding. Random selection of the same number of windows per dyad reproduces the observed mixture evidence, and no value of *k* from 1 to 10 exceeds its own matched null. We therefore present the mode positions as a description of where the model placed its attention rather than as detected temporal organization. The hard top-*k* constraint fixes how many decisive windows are selected but not where they fall, so the circularity lies not in the architecture but in the inference procedure applied to its output.

Two aspects of this null result warrant emphasis. First, the architecture does not encode phase boundaries. DXSP is free to place its *k* = 3 attention peaks anywhere in the interaction, so the failure to recover clustering is a property of the data and of the inference procedure rather than a constraint imposed by the model. Second, the null applies to the mixture evidence and not to every temporal analysis reported here. The phase analysis in Section 3.2 contrasts successful and failed dyads against each other rather than against a fixed expectation, and it does not depend on the mixture fit. Reporting the calibration alongside the fit is what allows these two levels of evidence to be separated.

### 4.2 Phase-outcome asymmetry and early engagement

The second principal finding is that the relative concentration of decisive moments across phases differs between successful and failed persuasion outcomes. Successful dyads concentrate 49.1% of their decisive moments in the opening phase. Failed dyads concentrate 42.0% in the middle phase. This asymmetry is supported by three converging statistical tests. The aggregate chi-square test, the within-dyad permutation test, and the dyad-level Mann–Whitney *U* test on the opening phase fraction all reach significance at the 0.05 threshold. Only the opening-phase comparison survives FDR correction. The Cramér *V* interval of 0.141 to 0.488 is wide, and its lower bound sits just above the conventional threshold for a small effect. With 19 successful and 27 failed dyads the estimate is imprecise. The sweep over *k* shows the association reaching significance at *k* = 3 but not at other values of *k*. We therefore treat this asymmetry as preliminary.

The interpretation of this asymmetry can be situated within existing theory in interpersonal communication. Successful persuasion has often been linked to early rapport establishment. The opening phase of an interaction sets the tone, establishes mutual orientation, and signals the participants willingness to engage. Decisive gesture moments concentrated in the opening phase may reflect this rapport-building process. Conversely, when decisive moments concentrate in the middle phase, the participants may be engaged in unresolved disagreement or in repeated attempts to find common ground. The middle-phase concentration in failed dyads is consistent with prolonged struggle to align rather than smooth progress toward persuasion.

This interpretation aligns with the literature on conversational alignment and accommodation. Pickering and Garrod (2004) describe how successful communication depends on interlocutors aligning at multiple linguistic levels. Early alignment cascades to facilitate subsequent communication. The opening-phase concentration of gesture peaks in successful dyads may represent an embodied counterpart of this early alignment. The pattern complements recent observations on interpersonal physiological synchrony, where context and individual factors shape synchrony emergence (Boukarras et al., 2025). The finding also extends prior text-based and audio-based alignment research to the gestural domain.

### 4.3 Positional structure and outcome content

The central contribution of this study is not outcome classification but the localization of decisive moments and the analysis of where those moments fall. A theoretical implication emerges from the contrast between the temporal-structural finding and the modest outcome-prediction performance of DXSP. This is not specific to DXSP. As Table 2 shows, no architecture we tested separates outcome above chance, which suggests the limitation lies in what gesture alone carries about outcome at this sample size rather than in the model. However, the same model produces attention positions that differentiate successful from failed dyads in their phase distribution. This contrast suggests that the model captures positional structure, corresponding to when persuasion-relevant moments occur, but does not directly capture content structure, corresponding to what is special about those moments.

This positional content distinction has several implications. From a theoretical perspective, it suggests that the temporal organization of persuasive interaction is a structural property that can be detected from gesture dynamics. The content at those moments, by contrast, may require additional modalities such as verbal content or facial expression to fully characterize (Krumhuber et al., 2023; Hu et al., 2024). From a practical perspective, the distinction implies that gesture-only systems may identify when to intervene in a persuasive interaction even without precise classification of individual interaction outcomes. A system can detect that an interaction is in its opening phase and prioritize rapport-building responses without needing to predict the final persuasion outcome.

This perspective reframes the modest AUC not as a failure of the approach but as a clarification of its proper use. DXSP is better described as a localizer of candidate decisive moments than as an outcome classifier. The positions it produces are what make the phase-level comparison in Section 3.2 possible. They do not need to predict outcome directly in order to be useful for that comparison.

### 4.4 Joint-level findings and design implications

The joint-level analysis shows the largest mean elevation for the nose joint, followed by the left wrist, although these differences do not survive correction across the seven joints. The nose joint primarily tracks head orientation in three-dimensional space when projected to a two-dimensional image plane. If this ranking were confirmed on a larger sample, it would be consistent with prior work emphasizing head movement and gaze direction as markers of engagement in interaction (Bohus and Horvitz, 2010). We do not treat the present result as support for that link. Importantly, head orientation can be tracked without the privacy concerns associated with facial expression analysis. Head orientation reveals orientation, not identity.

The left wrist contribution exceeds the right wrist contribution by 0.13 normalized units in mean velocity magnitude at decisive moments. This comparison was not tested statistically and is subject to the same limitation. This asymmetry may reflect handedness effects given the majority right-handed population, where the non-dominant left hand might engage in more expressive gestures while the dominant right hand engages in more instrumental or pointing gestures. Alternatively, the asymmetry may reflect cultural factors specific to the Japanese participants studied here. Distinguishing these explanations requires future work with larger samples and explicit handedness measurement.

These joint-level observations are exploratory and do not yet support design recommendations. If replicated on a larger sample, they would point toward the following directions. First, sensors tracking head orientation can provide a robust and privacy-preserving signal for engagement detection. Second, two-handed gesture tracking is preferable to one-handed tracking given the asymmetric contribution patterns. Third, attention to upper-body joints in general, rather than to facial micro-expressions, may be sufficient to detect persuasion-relevant structural moments. These guidelines are consistent with recent work on privacy-preserving sensing (Kumawat and Nagahara, 2022; Wang et al., 2023; Gu et al., 2023).

### 4.5 Implications for persuasive AI

The findings of this study have several implications for the design of persuasive AI systems. We highlight three implications.

First, intervention timing may benefit from awareness of interaction phase. The opening phase of an interaction appears to be a window for establishing the conditions of successful persuasion. Persuasive AI systems could detect when an interaction is in its opening phase and prioritize responses that build rapport, establish mutual orientation, and signal engagement. This phase-aware timing contrasts with content-agnostic interventions that ignore the temporal organization of the interaction. Recent surveys of persuasive technology emphasize this shift toward context-aware adaptive systems (Rogiers et al., 2024).

Second, response to resistance may be calibrated to interaction phase. When decisive moments concentrate in the middle phase, the interaction may be in a resistance-driven configuration. Persuasive AI systems could detect this configuration and respond with strategies that address resistance directly rather than continuing to deploy arguments that have already failed to land. Mid-phase concentration may signal the need for a strategy shift rather than for redoubled effort.

Third, privacy-preserving deployment is supported by gesture-only signals. The findings suggest that gesture-only analysis carries information about where decisive moments fall relative to interaction phase without recourse to facial expression or voice analysis. This supports deployment in privacy-sensitive contexts where face and voice signals are not available or not permitted. Examples include medical settings with privacy regulations, public spaces with bystander concerns, and educational contexts with student data protections (Kumawat and Nagahara, 2022). This claim is limited to the inference stage. A deployed system would still require a camera, and the video stream at the sensing stage carries the same identifiability as any other recording. What gesture-only analysis removes is the need to store, transmit, or process facial and vocal content downstream of pose extraction. Systems for which the camera itself is the primary concern would require additional measures such as on-device extraction with immediate discarding of frames.

### 4.6 Limitations

This study has several limitations that frame the interpretation of its findings.

First, the sample size of 46 dyads is modest. The phase-outcome contrast reached significance at *k* = 3, but the effect size estimate is imprecise and the study is underpowered for smaller effects or for interaction effects. Future work should replicate the findings on larger samples and on more demographically diverse populations.

Second, the corpus is narrow in two respects. All participants were Japanese university students, and all dialogues followed a single task structure built on low-stakes everyday scenarios. The cultural specificity of persuasive interaction patterns is well-documented (Funk et al., 2024). The phase-level patterns may generalize across cultures because they derive from organizational properties of persuasive discourse, but the specific timing of opening, middle, and closing peaks may shift in other cultural contexts. High-stakes persuasion in medical, political, or commercial settings may likewise exhibit different temporal patterns. Future work should examine both dimensions with comparable methods.

Third, the outcome variable was self-reported. Self-report measures of persuasion are subject to demand characteristics, social desirability bias, and limited introspective access. Future work should incorporate behavioral outcome measures such as decision changes, follow-through behaviors, or third-party judgments. Multimodal outcome measurement would strengthen confidence in the validity of the persuasion success label.

Fourth, the gesture-only design excludes potentially informative modalities. Verbal content, prosodic features, and facial expressions all carry persuasion-relevant information (Hu et al., 2024; Ramaswamy and Palaniswamy, 2024). The present study deliberately focuses on gesture alone for privacy and robustness motivations. Future work integrating multiple modalities would clarify the relative contribution of each modality and the boundary of what can be detected from gestures alone.

Fifth, the DXSP architecture uses a fixed hyperparameter *k* = 3. The results report a sweep over *k* from 1 to 10 but *k* remains fixed across dyads in the model itself. Whether the number of decisive moments should vary with the interaction rather than being imposed is untested.

Sixth, all recordings were made in a dedicated laboratory room with controlled lighting, a neutral background, and fixed camera placement. These conditions were chosen so that the analysis would reflect gesture dynamics rather than pose estimation failure. They also mean that the robustness of the pipeline under uncontrolled acquisition, including variable lighting, cluttered backgrounds, oblique viewing angles, and partial occlusion, remains to be established.

### 4.7 Future work

One direction is cross-cultural extension. We have collected a parallel corpus of Thai dyadic persuasion dialogues using the same recording protocol and the same persuasion task structure. Applying DXSP to the Thai corpus and comparing the inferred mode positions and phase outcome patterns would test the cross-cultural generalization of the present findings. Differences between Japanese and Thai patterns would identify culturally specific aspects of persuasive interaction structure. Such comparison is timely given recent attention to cross-cultural non-verbal behavior modeling (Funk et al., 2024).

A second direction is multimodal integration. Combining gesture analysis with verbal content analysis or audio-prosodic analysis would test whether the positional content distinction holds across modalities. Recent multimodal surveys identify multimodal fusion as a key open problem (Hu et al., 2024; Liang et al., 2024). If verbal content can predict outcome where gesture cannot, the distinction is modality-specific. If neither modality individually predicts outcome but their combination does, the distinction may reflect the need for multimodal evidence.

A third direction is real-time deployment. The DXSP architecture is causal at the window level and could be adapted for online inference. A real-time version of DXSP could be embedded in a conversational agent or coaching system to detect interaction phase and adapt strategy in real time. Evaluating such a system would test the practical utility of the present findings beyond their theoretical and statistical support. The present pipeline was evaluated offline, and end-to-end latency under streaming conditions, including the pose estimation stage, has not been measured. Establishing real-time feasibility would require that measurement on the target hardware.

A fourth direction is integration with verbal alignment frameworks. Pickering and Garrod (2004) describe linguistic alignment as a hierarchical process. Examining whether gestural decisive moments coincide with linguistic alignment events would test the integration of gesture and language in dyadic communication. This integration would extend the theoretical scope of the present findings.

## 5 Conclusion

We introduced DXSP, a gesture-only neural architecture for identifying sparse decisive moments in dyadic persuasive interaction. The architecture combines cross-attention, a concordance gate, and a hard top-*k* attention mechanism that enforces structural sparsity by construction. We evaluated DXSP on 46 Japanese dyadic persuasion dialogues using upper-body pose data from the Sapiens Goliath foundation model (Khirodkar et al., 2024).

Three principal findings emerged. First, mixture evidence for three-phase structure in the selected positions does not survive calibration against a matched random-selection null, and this holds at every value of *k* from 1 to 10. Second, the relative concentration of decisive moments across phases differs between successful and failed persuasion outcomes. Successful dyads concentrate decisive moments in the opening phase. Failed dyads concentrate decisive moments in the middle phase. Three converging statistical tests support this finding with medium effect size. Third, joint-level differences did not survive correction across the seven joints and the ranking is reported as exploratory.

These findings give preliminary evidence that gesture-only analysis carries information about where persuasion-relevant moments fall. They do not show that it recovers three-phase organization. Outcome differentiation appears at the level of phase distribution rather than direct classification, and it remains preliminary at this sample size. If replicated, this pattern would support the design of privacy-preserving persuasive AI systems that adapt intervention timing to interaction phase. The positional content distinction suggests that gesture analysis is better described as a localizer of candidate moments than as a content classifier. This perspective opens directions for multimodal integration, cross-cultural extension, and real-time deployment in persuasive technology.

## Data Availability

The data supporting the findings of this study are not publicly available because they contain identifiable recordings of human participants and are subject to privacy restrictions under the informed consent process. The data cannot be shared upon request.
